# Prospective Randomized Phase II Study of Stereotactic Body Radiotherapy (SBRT) vs. Conventional Fractionated Radiotherapy (CFRT) for Chinese Patients with Early-Stage Localized Prostate Cancer †

**DOI:** 10.3390/curroncol29010003

**Published:** 2021-12-22

**Authors:** Darren M. C. Poon, Daisy Lam, Kenneth C. W. Wong, Cheuk-Man Chu, Michael Cheung, Frankie Mo, Joyce Suen, Chi-Fai Ng, Anthony T. C. Chan

**Affiliations:** 1 Department of Clinical Oncology, State Key Laboratory of Translational Oncology, Sir YK Pao Centre for Cancer, Hong Kong Cancer Institute and Prince of Wales Hospital, The Chinese University of Hong Kong, Hong Kong, China; lcm306@ha.org.hk (D.L.); wcw979@ha.org.hk (K.C.W.W.); clm314@ha.org.hk (M.C.); frankie@clo.cuhk.edu.hk (F.M.); j_suen@clo.cuhk.edu.hk (J.S.); anthonytcchan@cuhk.edu.hk (A.T.C.C.); 2 Comprehensive Oncology Centre, Hong Kong Sanatorium & Hospital, Hong Kong, China; 3 Department of Imaging and Interventional Radiology, Prince of Wales Hospital, The Chinese University of Hong Kong, Hong Kong, China; charmantchu@gmail.com or; 4 Department of Surgery, Prince of Wales Hospital, The Chinese University of Hong Kong, Hong Kong, China; ngcf@surgery.cuhk.edu.hk

**Keywords:** dose fractionation (radiation), patient reported outcomes, prostate cancer, quality of life, radiation tolerance, stereotactic body radiotherapy

## Abstract

Background: Stereotactic body radiotherapy (SBRT) has potential radiobiologic and economic advantages over conventional fractionated radiotherapy (CFRT) in localized prostate cancer (PC). This study aimed to compare the effects of these two distinct fractionations on patient-reported quality of life (PRQOL) and tolerability. Methods: In this prospective phase II study, patients with low- and intermediate-risk localized PC were randomly assigned in a 1:1 ratio to the SBRT (36.25 Gy/5 fractions/2 weeks) or CFRT (76 Gy/38 fractions/7.5 weeks) treatment groups. The primary endpoint of variation in PRQOL at 1 year was assessed by changes in the Expanded Prostate Cancer Index Composite (EPIC) questionnaire scores and analysed by z-tests and *t*-tests. Results: Sixty-four eligible Chinese men were treated (SBRT, *n* = 31; CFRT, *n* = 33) with a median follow-up of 2.3 years. At 1 year, 40.0%/46.9% of SBRT/CFRT patients had a >5-point decrease in bowel score (*p* = 0.08/0.28), respectively, and 53.3%/46.9% had a >2-point decrease in urinary score (*p* = 0.21/0.07). There were no significant differences in EPIC score changes between the arms at 3, 6, 9 and 12 months, but SBRT was associated with significantly fewer grade ≥ 1 acute and 1-year late gastrointestinal toxicities (acute: 35% vs. 87%, *p* < 0.0001; 1-year late: 64% vs. 84%, *p* = 0.03), and grade ≥ 2 acute genitourinary toxicities (3% vs. 24%, *p* = 0.04) compared with CFRT. Conclusion: SBRT offered similar PRQOL and less toxicity compared with CFRT in Chinese men with localized PC.

## 1. Introduction

External beam radiotherapy (EBRT) is an effective curative treatment option for localized prostate cancer (PC) [1]. Conventional fractionated radiotherapy (CFRT), with daily dose fractionation of 1.8–2 Gy over 8 to 9 weeks, has been commonly administered worldwide [2]. However, such a protracted total treatment time, together with the mounting incidence of PC, poses burdens for the healthcare system [3]. Epidemiological studies have estimated that, optimally, 60% of PC patients require radiotherapy (RT) at some point in their illness [4,5]. In a recent U.S. modeling study, for low-risk PC patients, RT is the most expensive initial treatment option, and results in the highest 10-year cumulative cost [6]. However, even in high-income countries, disparities in access to standard-of-care RT exist because of socio-geographical factors [7].

The unique radiobiologic characteristics of PC open new possibilities for shortening the overall radiation treatment time. In theory, a low alpha/beta ratio entails a more pronounced linear-quadratic dose response, with greater killing per unit dose at higher doses [8], i.e., an increased fraction sensitivity. The low alpha/beta ratio (range: 0.9–2.2) of PC, which has been reported across low-, intermediate- and high-risk patient groups [9,10], suggests that the therapeutic ratio could potentially be enhanced by hypofractionation. Clinical trials published in the past several years showed non-inferiority of moderately hypofractionated RT (MHRT; fraction size 2.4–3.4 Gy over 4 to 6 weeks) for biochemical disease-free survival, and similar toxicity compared with CFRT [11,12,13,14]. MHRT is now the recommended EBRT option [15].

With the emergence of high-precision RT techniques such as image guidance systems, further shortening of overall treatment times with stereotactic body RT (SBRT; 5–6 fractions of extremely high-dose radiation, ≥500 cGy per fraction over 2 to 3 weeks) is hypothetically feasible. Ample prospective, single-arm trials have demonstrated promising efficacy and favorable toxicity of SBRT that is largely comparable to CFRT. In the pooled analysis of multi-institutional prospective phase II studies, SBRT showed 5-year biochemical relapse-free survival (bRFS) rates of 93%, 95%, 84% and 81% for all, low-, intermediate- and high-risk patients, respectively [16]. In a recent meta-analysis including 6116 patients among 38 prospective studies, the overall 5- and 7-year bRFS rates were 95.3% and 93.7%, whereas the estimated late grade ≥ 3 genitourinary (GU) and gastrointestinal (GI) toxicity rates were 2.0% and 1.1%, respectively [17]. In another meta-analysis of 7 phase III trials in men with localized PC (*n* = 6795), the 5-year cumulative incidence of late grade ≥ 2 GU toxicity was comparable between ultrahypofractionated RT, hypofractionated RT and CFRT, at 18%, 20.4% and 19.4%, respectively (*p* = 0.92; random effects model) [18].

Two phase III trials of SBRT and CFRT were ongoing at the time of our study: PACE (NCT01584258) for low- or intermediate-risk PC patients (8% and 92%, respectively) and HYPO-RT-PC (ISRCTN45905321) in PC patients with intermediate and high risks (89% and 11%). The latest published results of HYPRO-RT-PC confirmed highly similar failure-free survival at 5 years (84% in both arms, hazard ratio = 1.002, log-rank *p* = 0.99) [19]. There was a small increase in early side-effects such as urinary toxicities in the SBRT group, but toxicity was otherwise similar at up to 5-year follow-up. For PACE-B, while efficacy results are not yet mature, short-term toxicity findings were similar between-arms: radiation therapy oncology group (RTOG) grade ≥ 2 GI toxicities were reported in 10% (SBRT) vs. 12% (CFRT; *p* = 0.38) and grade ≥ 2 GU toxicities in 23% vs. 27% of patients (*p* = 0.16), respectively [20].

While further efficacy and long-term safety results are needed, the growing body of evidence supports potential radiobiologic and economic benefits of SBRT for PC. Our present phase II study was designed to provide evidence in the form of a prospective, randomized trial evaluation and head-to-head comparison of the patient-reported quality of life (PRQOL) and treatment-related toxicities with SBRT vs. CFRT in low- and intermediate-risk localized PC.

## 2. Results

### 2.1. Demographic Characteristics

**Figure 1:**
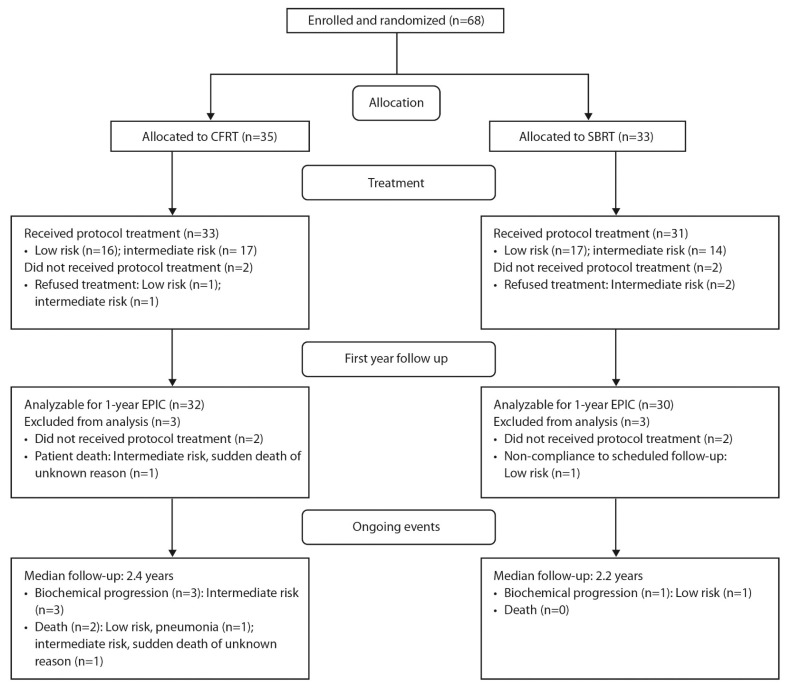
CONSORT diagram showing enrollment, random assignment and follow-up of the study participants. Abbreviations: SBRT, stereotactic body radiotherapy; CFRT, conventional fractionated radiotherapy; EPIC, Expanded Prostate Cancer Index Composite questionnaire.

Between January 2015 and May 2017, 68 patients were enrolled (Figure 1); four patients were ineligible. The baseline characteristics of 64 patients who received the protocol treatment with follow-up were well-balanced and are listed in Table 1. The median age was 69.5 years and the median pre-treatment prostate-specific antigen (PSA) was 8.1 ng/mL. In general, 93% had a Zubrod performance score of 0. National Comprehensive Cancer Network (NCCN) low- and intermediate-risk patients were equally represented in both arms.

### 2.2. Treatments Received

Of 64 eligible patients, 31 received SBRT and 33 received CFRT. Neoadjuvant androgen-deprivation therapy (ADT) was given in 10 patients (SBRT: 4; CFRT: 6). A total of 6 months of ADT with luteinizing hormone-releasing hormone agonists were prescribed 3 months prior to RT. Median follow-up from the beginning of RT was 2.2 (range: 1.7–2.7) and 2.4 (range: 1.8–2.9) years for the SBRT and CFRT arms, respectively. All 64 patients were analyzed, with no protocol violations, and none was lost to follow-up.

### 2.3. PRQOL

**Figure 2:**
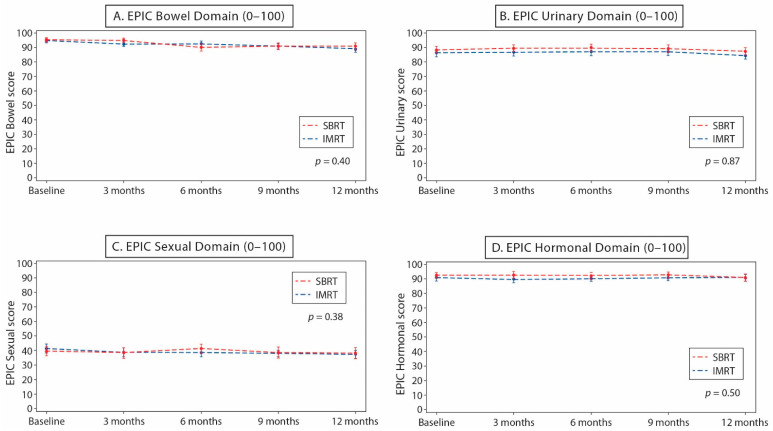
Mean (95% confidence interval) Expanded Prostate Cancer Index Composite questionnaire (EPIC) score over time for stereotactic body radiotherapy (SBRT) and conventional fractionated radiotherapy (CFRT): (**A**) bowel domain; (**B**) urinary domain; (**C**) sexual domain; and (**D**) hormonal domain. Abbreviations: EPIC, Expanded Prostate Cancer Index Composite questionnaire; SBRT, stereotactic body radiotherapy; CFRT, conventional fractionated radiotherapy.

The Expanded Prostate Cancer Index Composite (EPIC) questionnaire completion compliance rate was 100% (64/64) before treatment and 96.9% (62/64) at 1 year. Given the median follow-up of 2.2–2.4 years, the 2-year or later EPIC results will not be presented here. There were no significant differences in change of score between the arms with respect to the urinary and bowel, as well as the sexual and hormonal, EPIC domains at 3, 6, 9 and 12 months (Figure 2). At 1 year, 12 (40.0%) SBRT and 15 (46.9%) CFRT patients had a >5-point reduction in EPIC bowel score compared with baseline (SBRT, *p* = 0.28; CFRT, *p* = 0.08). Regarding the EPIC urinary domain, 16 (53.3%) SBRT and 15 (46.9%) CFRT patients had a >2-point score reduction at 1 year compared with baseline (SBRT, *p* = 0.07; CFRT, *p* = 0.21; Table 2). In the SBRT arm, compared with pre-treatment assessment, 9 patients (30%) had a >11-point reduction in 1-year EPIC sexual score compared with baseline (*p* = 0.28) and 13 patients (43%) had a >3-point reduction in 1-year EPIC hormonal score compared with baseline (*p* = 0.27). In the CFRT arm, eight patients (25%) experienced a >11-point reduction in EPIC sexual score at 1 year (*p* = 0.12) and eight patients (25%) had a >3-point reduction in 1-year EPIC hormonal score (*p* = 0.06).

### 2.4. Acute and Late Toxicities

There were no grade ≥ 3 acute toxicities reported in either arm. SBRT patients experienced significantly fewer ≥ grade 1 acute GI toxicities (cumulative number: 35% vs. 87%, *p* < 0.0001) and grade ≥ 2 acute GU toxicities (cumulative number: 3% vs. 24%, *p* = 0.0426) compared with CFRT patients. At the 1-year follow-up, two grade 3 GU late toxicities, one in each arm (SBRT: non-infective cystitis [3%]; CFRT: urinary incontinence [3%]), were reported. SBRT patients experienced significantly fewer grade ≥1 late GI toxicities (cumulative number: 64% vs. 84%, *p* = 0.033) and a similar rate of grade ≥ 1 late GU toxicities (cumulative number: 93% vs. 100%, *p* = 0.2307) than CFRT patients (Table 3).

### 2.5. Disease Control

At 1 year, two patients in the CFRT group had died of diseases unrelated to their PC (community-acquired pneumonia and sudden death of unknown reason). The overall survival rates at 1 year for the whole cohort, SBRT and CFRT patients were 98.4%, 100% and 97%, respectively (*p* = 0.08). Biochemical progressions (Phoenix criteria, PSA nadir + 2 ng/mL [21]) occurred in two CFRT patients, resulting in 98.4%, 100% and 97% biochemical failure-free survival at 1 year for all patients, SBRT and CFRT groups, respectively (*p* = 0.08).

## 3. Discussion

In this phase II study of SBRT vs. CFRT in low- and intermediate-risk localized PC, SBRT resulted in a similar PRQOL in terms of the proportion of patients with significant reductions in EPIC bowel and urinary scores at 1 year from baseline, and seemingly favorable physician-scored acute and late toxicities compared with CFRT. Our results suggest that SBRT is a safe, tolerable alternative to CFRT for patients with early-stage localized PC.

MHRT, based on non-inferiority to CFRT in several randomized landmark studies, is currently the recommended EBRT option for localized PC [15], but was not recognized as such when our study was conceived. In comparison to CFRT, SBRT offers similar benefits to MHRT in terms of patient convenience and resource utilization, with much shorter travel and treatment times, and potentially higher cost-effectiveness [22].

Even more important in establishing the role of SBRT in managing localized PC is its safety and tolerability. Whereas HYPO-RT-PC observed higher levels of self-reported acute urinary and bowel symptoms in patients receiving SBRT vs. CFRT, PACE-B did not (or even found slightly less acute toxicity in the SBRT arm). This might have been due to (i) the inclusion of high-risk patients in HYPO-RT-PC and low-risk patients in PACE-B, (ii) an SBRT dosage difference (HYPO-RT-PC: 42.7 Gy/7 fractions [frs]/2.5 weeks vs. PACE-B: 36.25 Gy/5 frs/1–2 weeks) and/or (iii) the majority (70%) of controls in PACE-B receiving MHRT (62 Gy/20 frs/4 weeks). The present study found lower levels of GI and GU toxicity in patients receiving SBRT vs. CFRT, with more pronounced differences that could be attributable to an underpowered sample size.

Our results corroborate previous reports with regard to the favorable physician-scored toxicity of SBRT in localized PC, with <5% acute and late grade ≥ 3 GI and GU complications [17]. Aside from similar 1-year late GU toxicities between the two arms, fewer grade ≥ 1 acute/1-year late GI and grade ≥ 2 GU toxicities were reported with SBRT in our study. Interestingly, there was disagreement between patient-reported outcomes (PROs) and physician-scored toxicities, with seemingly favorable side effects with SBRT but similar PROs between the two treatment arms. This highlights the well-known challenges in assessing treatment-related outcomes, particularly PROs, in open-label cancer trials [23,24,25]. By integrating both PROs and physician-scored toxicities collectively, our study demonstrated that SBRT is not inferior to CFRT in treating localized PC.

Relatively few studies assessed the tolerability and efficacy of SBRT in intermediate-risk compared with low-risk PC. Half of our study involved intermediate-risk patients, demonstrating that SBRT is feasible and comparable to CFRT in this subgroup. Nonetheless, because we included the seminal vesicles (SVs) for irradiation (Supplementary Methods), the risk of possible adverse consequences with SBRT in the intermediate-risk group patients may be higher. The recent RTOG-0938 study [26], which evaluated two regimens of ultra-hypofractionation (36.25 Gy/5 frs/2 weeks and 51.6 Gy/12 frs/2 weeks) for low-risk PC without incorporation of SVs in the high-dose zone, reported a seemingly lower proportion of EPIC score decline in patients randomized to the SBRT arm (36.25 Gy/5 frs/2 weeks) than ours. Specifically, only 29.8% of their SBRT patients had a >5-point reduction in EPIC bowel score from baseline at 1 year, compared with 40.0% of our SBRT patients. Similarly, 45.7% of their patients and 53.3% of our patients had a >2-point reduction in 1-year EPIC urinary score from baseline. Although a cross-trial comparison would be inappropriate statistically, the numerical difference in the proportion of patients with EPIC score decline between the two studies may be partly attributed to the SV irradiation in the intermediate-risk group. However, the degree of EPIC deterioration was similar between SBRT and CFRT in this study, suggesting that such an influence is more likely related to the larger irradiation volume than the dose-fractionation in intermediate-risk disease.

This study has several limitations. First, this trial only compared SBRT to CFRT, and not the currently recommended MHRT. However, since various studies have established the non-inferiority of MHRT to CFRT, we expect that the results of a SBRT vs. MHRT comparison will be similar. Second, the limited data on long-term PROs and efficacy can be attributed to the relatively short follow-up. While follow-up of our cohorts will continue, previous retrospective series and pooled-analysis demonstrated comparable long-term efficacy and tolerability to other definitive treatments, suggesting that our preliminary results will likely be sustained [16,17,27]. Third, endorectal balloons (ERBs) were used only in the SBRT arm, thus their potential benefits, e.g., reducing intra-fractional prostatic motion and displacing the posterior part of the rectum out of the high-dose zone, could have contributed to the better tolerability in the SBRT arm. However, such benefits could have been outweighed by the considerable detrimental dosimetric effect of ERBs on the rectum via the displacement of the anterior rectal wall into the ultra-high-dose zone with SBRT, which was shown in a prior dosimetric study [28]. Furthermore, 7–8 weeks of daily ERB application in the CFRT arm would have been impractical and disturbing for the patients.

## 4. Materials and Methods

### 4.1. Trial Design

This was a single-institution, unblinded randomized phase II study with 1:1 random assignment to SBRT (36.25 Gy in 5 frs over 2 weeks) or CFRT (76 Gy in 38 frs over 7.5 weeks). Participants were randomly assigned by the minimization method to either SBRT or CFRT, and stratified by the risk of localized PC using the NCCN risk classification (low vs. intermediate). The study was approved by the institutional review board (CUHK/NTEC CREC Ref. No. 2013.483-T) and registered at ClinicalTrials.gov (NCT02339701).

### 4.2. Study Patients

Men aged ≥ 18 years with a histologic diagnosis of prostate adenocarcinoma and NCCN low- or intermediate-risk (T1-2, Gleason score ≤ 7 and PSA < 20 ng/mL) localized disease were eligible for the study. Additional criteria were Zubrod performance status < 2, no nodal or distant metastasis, and no prior bilateral orchiectomy, chemotherapy, RT, cryosurgery, or definitive surgery for PC. Patients with another invasive cancer, other than localized basal or squamous cell skin carcinoma, were ineligible. Only patients who were willing and able to complete the EPIC questionnaire and signed and understood the informed consent were enrolled.

### 4.3. Treatments

The prescription doses to planning target volume (PTV)-1/PTV-2 in the SBRT and CFRT arms were 36.25 Gy/32.5 Gy in five frs and 76 Gy/70 Gy in 38 frs, respectively. The patients were treated with volumetric modulated arc therapy (VMAT) using a Varian TrueBeam 2.0 linear accelerator with Millennium 120 MLC (Supplementary Methods). Dose constraints to normal tissues (bladder, rectum, penile bulb) were as listed in the protocol (in Supplementary Materials). Neoadjuvant androgen-deprivation therapy (ADT) was optional, given at physician’s discretion for intermediate-risk PC patients.

### 4.4. Patient Assessments

At baseline, patient history, physical examination, toxicity and performance status were assessed. Pre-treatment assessment also included PSA measurement and the EPIC questionnaire [29]. The serine protease PSA is almost produced exclusively by prostate epithelial cells, and it is thought that prostate tumor growth leads to leakage of PSA into the blood [30]. Early-stage PC tumors secrete PSA, which is useful as a biomarker for monitoring response to therapy, and predicting pathologic stages over time [30].

In this study, the traditional Chinese version of EPIC was used (see Clinical Trial Protocol in Supplementary Materials), which was translated and culturally adapted from the original English version in a validation study [31]. Both versions of the instrument are now available on the University of Michigan Department of Urology website (https://medicine.umich.edu/dept/urology/research/epic, accessed on 17 December 2021).

Patients were evaluated weekly during RT for performance status and toxicities. An acute adverse event was defined as the first occurrence of worst severity of the adverse event from the beginning of RT until ≤30 days after the completion of RT. Both acute and late adverse events were evaluated with the Common Terminology Criteria for Adverse Events (version 4.0). Assessments were performed and the EPIC questionnaire collected every 3 months for the first 2 years, every 6 months for the next 3 years and annually thereafter.

### 4.5. Study Endpoints

The primary endpoint of this study was to evaluate and compare the PRQOL by the proportion of patients with >5-point and >2-point reductions in the EPIC bowel and urinary domains, respectively, at 1 year compared with baseline, between the two treatment arms. Additional endpoints included the sexual and hormonal EPIC scores, acute and late toxicities, bRFS, and overall survival.

### 4.6. Statistical Analysis

As in the RTOG 0415 and RTOG 0938 studies, the proportion of patients with a change in EPIC bowel domain score (baseline to 1-year) worse than 5 points and a change in urinary domain score worse than 2 points were considered to be clinically meaningful endpoints for the tolerability and safety of radical prostate RT [12,26]. These thresholds were selected from an analysis of EPIC scores in 108 patients who received standard RT treatment in RTOG 0415, based on the universal notion that half of a standard deviation constitutes a threshold of discrimination for changes in health-related QOL for chronic diseases [26,32]. EPIC change scores were compared between treatment arms using a *t*-test. *p*-values < 0.05 were considered statistically significant. All analyses were conducted using Statistical Analysis Software (SAS for Windows, version 9.3).

## 5. Conclusions

SBRT had similar PRQOL and less toxicity than CFRT in this phase II study of Chinese men with localized PC. In corroboration with the latest phase III results, our results support SBRT as a safe and tolerable treatment option in low- and intermediate-risk PC.

## Figures and Tables

**Table 1:** Patient baseline characteristics.

Patient Characteristics	SBRT (*n* = 31)	CFRT (*n* = 33)
Age		
Mean (SD)	69.4 (6.0)	69.0 (6.8)
Median (range)	68 (53–78)	70 (55–81)
Zubrod Performance		
0	30 (96%)	30 (90%)
1	1 (3%)	3 (10%)
Clinical T Stage		
1a	1 (3%)	0
1c	16 (51%)	15 (45%)
2a	7 (22%)	10 (30%)
2b	5 (16%)	3 (9%)
2c	2 (6%)	5 (15%)
Gleason Score		
5	3 (9%)	0
6	16 (51%)	22 (66%)
7	12 (38%)	11 (33%)
PSA		
Mean (SD)	9.2 (5.0)	8.6 (5.4)
Median (Q1–Q3)	8.8 (6.0–11.8)	7.6 (5.8–10.3)
NCCN Risk Group		
Low	16 (51%)	16 (48%)
Intermediate	15 (48%)	17 (51%)

Abbreviations: SD, standard deviation; PSA, prostate-specific antigen; Q1–Q3, first to third quartile; NCCN, National Comprehensive Cancer Network risk classification; SBRT, stereotactic body radiotherapy; CFRT, conventional fractionated radiotherapy.

**Table 2:** Patient-reported quality of life: Expanded Prostate Cancer Index Composite questionnaire (EPIC) score change at 1 year from baseline.

Domain	SBRT	*p*-Value	CFRT	*p*-Value
Bowel				
Patients, no.	30		32	
Mean (SD)	−4.2 (12.5)	0.40 **	−5.8 (9.9)	
Median	0.0		−1.8	
>5-point reduction, no (%) ^†^	12 (40%)	0.28 *	15 (46.9%)	0.08 *
Urinary				
Patients, no.	30		32	
Mean (SD)	−1.3 (12.9)	0.87 **	−2.3 (12.7)	
Median	−2.1		0.0	
>2-point reduction, no (%) ^‡^	16 (53.3%)	0.07 *	15 (46.9%)	0.21 *
Sexual				
Patients, no.	30		32	
Mean (SD)	−1.9 (15.3)	0.38 **	−3.8 (18.3)	
Median	0.3		−1.8	
>11-point reduction, no (%) ^§^	9 (30%)	0.28 *	8 (25%)	0.12 *
Hormonal				
Patients, no.	30		32	
Mean (SD)	−1.3 (13.8)	0.50 **	0.2 (13.6)	
Median	0.0		0.0	
>3-point reduction, no (%) ^¶^	13 (43%)	0.27 *	8 (25%)	0.06 *

Abbreviations: EPIC, Expanded Prostate Cancer Index Composite questionnaire; SD, standard deviation; SBRT, stereotactic body radiotherapy; CFRT, conventional fractionated radiotherapy. * *p*-value from one-sided, one-sample z-test (before vs. after). ** *p*-value for the comparison between SBRT and CFRT. ^†^ Rate ≤ 35%, acceptable; rate ≥ 60%, unacceptable (see Statistical Analysis section in Methods on details of the acceptability/unacceptability thresholds). ^‡^ Rate ≤ 40%, acceptable; rate ≥ 65%, unacceptable. ^§^ Rate ≤ 35%, acceptable; rate ≥ 60%, unacceptable. ^¶^ Rate ≤ 38%, acceptable; rate ≥ 63%, unacceptable.

**Table 3:** Acute and 1-year late gastrointestinal (GI) and genitourinary (GU) adverse events according to treatment assignment.

Adverse Event (Maximum Grade)	SBRT (*n* = 31)	CFRT (*n* = 33)	*p*-Value
Acute GI Toxicity			
None reported	20 (64%)	4 (12%)	*p* < 0.0001 ^†^
1	9 (29%)	22 (66%)	
2	2 (6%)	7 (21%)	
≥3	0	0	
≥1 (Total)	11 (35%)	29 (87%)	
Acute GU Toxicity			
None reported	3 (9%)	0	*p* = 0.0426 ^‡^
1	26 (83%)	25 (75%)	
2	1 (3%)	8 (24%)	
≥3	0	0	
≥1 (Total)	27 (87%)	33 (100%)	
1-Year Late GI Toxicity			
None reported	11 (35%)	5 (15%)	*p* = 0.033 ^†^
1	16 (51%)	22 (66%)	
2	4 (12%)	6 (18%)	
≥3	0	0	
≥1 (Total)	20 (64%)	28 (84%)	
1-Year Late GU Toxicity			
None reported	2 (6%)	0	*p* = 0.2307 ^†^
1	23 (74%)	25 (75%)	
2	5 (16%)	7 (21%)	
≥3	1 (3%)	1 (3%)	
≥1 (Total)	29 (93%)	33 (100%)	

Abbreviations: Gastrointestinal (GI) toxicity, toxicities including abdominal pain, bloating, constipation, diarrhea, fecal incontinence, hemorrhoids, proctitis, rectal hemorrhage, and rectal pain; genitourinary (GU) toxicity, toxicities including non-infective cystitis, hematuria, urinary frequency, urgency, retention, incontinence, and urinary tract pain; SBRT, stereotactic body radiotherapy; CFRT, conventional fractionated radiotherapy. ^†^ *p* for comparison of treatment group of grade ≥ 1 vs. grade < 1; ^‡^ *p* for comparison of treatment group of grade ≥ 2 vs. grade < 2.

## Data Availability

Request to obtain the study data may be addressed to the corresponding author. The data are not publicly available due to ethical (privacy) and legal considerations.

## Supplementary Materials

The following are available online at https://www.mdpi.com/article/10.3390/curroncol29010003/s1, Supplementary Methods and Clinical Trial Protocol.
