# Supplementary material for: Prospective Randomized Phase II Study of Stereotactic Body Radiotherapy (SBRT) vs. Conventional Fractionated Radiotherapy (CFRT) for Chinese Patients with Early-Stage Localized Prostate Cancer
Source: Curr Oncol. 2021 Dec 22;29(1):27–37. doi: 10.3390/curroncol29010003 (PMC8774487; doi:10.3390/curroncol29010003)
Supplement: Supplementary file 1 [file curroncol-29-00003-s001.zip › Clinical Trial Protocol.pdf]

Protocol Title

A randomized, open label, phase II study: stereotactic body radiotherapy vs. conventional intensity-modulated radiotherapy for low- or intermediate-risk prostate cancer in an Asian population

Institutions

Department of Clinical Oncology
The Chinese University of Hong Kong
Prince of Wales Hospital
Hong Kong

Department of Imaging and Interventional Radiology
The Chinese University of Hong Kong
Prince of Wales Hospital
Hong Kong

Department of Surgery,
The Chinese University of Hong Kong
Prince of Wales Hospital
Hong Kong

Investigators

Principal investigator:

Dr. Darren M.C. Poon
Specialist, Department of Clinical Oncology
The Chinese University of Hong Kong
Prince of Wales Hospital

Co-investigators:

Dr. Michael K.M. Kam,
Consultant, Department of Clinical Oncology,
The Chinese University of Hong Kong,
Prince of Wales Hospital

Dr. Florence S.T. Mok
Specialist, Department of Clinical Oncology
The Chinese University of Hong Kong
Prince of Wales Hospital

Dr. C.M. Chu
Department of Imaging and Interventional Radiology
The Chinese University of Hong Kong
Prince of Wales Hospital

Prof. Anthony CF Ng
Professor, Department of Surgery,
The Chinese University of Hong Kong,
Prince of Wales Hospital

Statistician

Frankie Mo
Department of Clinical Oncology,
The Chinese University of Hong Kong,
Prince of Wales Hospital

Version No.: 1.0
Version date: 09 Sep 2013

Protocol Summary

Title

A randomized, open label, phase II study: stereotactic body radiotherapy vs. conventional intensity-modulated radiotherapy for low- or intermediate-risk prostate cancer in an Asian population

Objectives

Primary Objective

- To compare the 1-year health-related quality of life (HRQOL) between SBRT and IMRT as measured by the Bowel and Urinary domains of the Expanded Prostate Cancer Index Composite (EPIC) instrument.

Secondary Objectives

- To estimate the rate of acute and late GI and GU toxicity for each arm at 1, 2, and 5 years
- To estimate biochemical-failure free survival in each arm at 1, 2, and 5 years
- To estimate disease-specific survival in each arm at 1, 2, and 5 years
- To estimate overall survival in each arm at 1, 2, and 5 years

Design

**Stratify**	Risk group<br>1. Low risk<br>2. Intermediate risk	**Randomize**	**Arm 1**<br>Conventional IMRT<br>76Gy in 38 fractions of 2 Gy over 7.5 weeks
			**Arm 2**<br>SBRT<br>36.25Gy in 5 fractions of 7.25Gy, twice a week over 15-17 days

Patient population

Patients with low or intermediate risk (i.e. T1-T2c and PSA < 20 and Gleason score <8) clinically localized prostate cancer

Index

1.0 Introduction
2.0 Objectives
3.0 Patient selection
4.0 Pre-treatment evaluation and management
5.0 Radiotherapy
6.0 Hormonal therapy
7.0 Patient assessment
8.0 Statistical consideration
9.0 Data and protocol management
10.0 Publication policy

References
Appendix I– Study parameters
Appendix II– Performance status scoring
Appendix III– staging system
Appendix IV –EPIC questionnaire (traditional Chinese version)

Title

A randomized, open label, phase II study: stereotactic body radiotherapy vs. conventional intensity-modulated radiotherapy for low- or intermediate-risk prostate cancer in an Asian population

1.0 Introduction

Radiotherapy is a common treatment alternative for prostate cancer and patients are commonly treated daily over a period from 7-8weeks. Recent analysis of the tumour biology of prostate cancer has suggested that it is more sensitive to larger dose per fraction (1-5). Appropriately designed schedules using large fractions (i.e. hypofractionated regimen) could result in increases in biochemical control with no increase in late sequelae. In addition to the potential for significant therapeutic gain, if a hypofractionated regimen is found to produce comparable findings it would result in substantial health care cost savings and would also be more convenient for patients.

Results of those hypofractionated randomized trial came from Canada and has been published (8). The trial compared 66 Gy/33 fractions (Long arm) to 52.5 Gy/20 fractions(Short arm) in men with low- and intermediate-risk prostate cancer.In this trial the 5-year rate of failure (biochemical or clinical) was higher in the Short arm compared to the Long arm (59.95% vs. 52.95%; HR 1.18 [0.99-1.41], p < 0.05). At first glance this would appear to suggest that the hypofractionated regimen may be inferior compared to a conventionally fractionated regimen, but the two arms were not designed to be isoeffective. Infact, using the biologically effective dose, the Short arm is consistently less than the Long arm until the alpha-beta ratio reaches a value of < 1. The results of the Canadian trial, therefore, are not inconsistent with an alpha-beta ratio for prostate cancer of 1.5. At a median follow-up of 5.7years there was no difference in 5-year actuarial rate of late grade 3+ GI/GU toxicity between the two arms.

In recent years investigators have looked at even shorter radiotherapy schedules (i.e. Stereotactic body radiotherapy (SBRT)). Stanford reported on 41 low risk patients treated with 36.25 Gy in 5 fractions using stereotactic body radiotherapy (6-7). The first 21 patients were treated on consecutive days but, due to higher than expected rectal toxicity treatments were modified to be given every other day (three fractions a week). With these changes no patients experienced Grade 3 or higher toxicity.

The below table summarizes the published SBRT studies (6-7, 9-12), all of which are single-arm phase I/II trials, and most of which involve <100 patients. Toxicity appears acceptable, having consisted primarily of grade 2 complications and grade 3 complications are rare. The biochemical control looks promising in these studies.

Author	Sample size	Median follow-up (Months)	Regimen	Grade 2 or above toxicity	Freedom from biochemical

				(%)	failure (%)
Madsen	40	41	33.5Gy/6.7Gy	GU: 20<br>GI: 7.5	90
King	67	32	36.25Gy/7.25Gy	GU: 8.5<br>GI: 2	94
Friedland	112	24	35Gy/7Gy	<10	>95
Katz	304	30	36.25Gy/7.25Gy	<10	>95
Mcbride	45	44.5	37.5Gy/7.5Gy	GU: !9<br>GI: 12	98
Boike	45	30	45Gy/9Gy	GI: 18<br>GU: 31	100

The efficacy and tolerability of SBRT for the Chinese prostate cancer patients remains unresolved. As the incidence of prostate cancer is mounting, it is beneficial, from the cost-effectiveness point-of-view, to investigate the feasibility of shorter radiotherapy regimen in Chinese prostate cancer patients, in which more patients can be treated within the same period of time and the travelling time to radiotherapy centre (from 7-8 weeks to 2 weeks) would be largely reduced.

2.0 Objectives

Primary Objective

- To compare the 1-year health-related quality of life (HRQOL) between SBRT and IMRT as measured by the Bowel and Urinary domains of the Expanded Prostate Cancer Index Composite (EPIC) instrument.

Secondary Objectives

- To estimate the rate of acute and late GI and GU toxicity for each arm at 1, 2, and 5 years
- To estimate biochemical-failure free survival in each arm at 1, 2, and 5 years
- To estimate disease-specific survival in each arm at 1, 2, and 5 years
- To estimate overall survival in each arm at 1, 2, and 5 years

3.0 Patient Selection

Patients from the both the Department of Clinical Oncology and Surgery are potential candidates to be recruited into this study.

Conditions for Patient Eligibility
- Histological confirmation of prostate adenocarcinoma
- Low or intermediate risk prostate cancer patients (i.e. T1-T2c and PSA < 20 and Gleason score < 8) with the risk of pelvic node metastasis ≤ 15% as calculated by Roach’s formula
- ECOG performance score 0-1
- Age ≥ 18
- History/physical examination within 2 weeks prior to registration
- Able to sign informed-consent

Conditions for Patient Ineligibility

- Patients with previous diagnosis of cancer other than prostate cancer and non-melanoma skin cancer.
- Evidence of distant metastases
- Regional lymph node involvement
- Previous radical surgery (prostatectomy), cryosurgery, or HIFU for prostate cancer
- Previous pelvic irradiation, prostate brachytherapy, or bilateral orchiectomy
- Previous hormonal therapy, such as LHRH agonists (e.g., goserelin, leuprolide) or LHRH antagonists (e.g., degarelix), anti-androgens (e.g., flutamide, bicalutamide), estrogens (e.g.DES), or surgical castration (orchiectomy)
- Unstable angina and/or congestive heart failure requiring hospitalization, transmural myocardial infarction within the last 6 months, acute bacterial or fungal infection requiring intravenous antibiotics, chronic obstructive pulmonary disease exacerbation or other respiratory illness requiring hospitalization or precluding study therapy at the time of registration
- Patients who have received prior chemotherapy.

4.0 Pre-treatment evaluation and management

Patients recruited into this study are required to complete the investigation including MRI pelvis. Bone scan will be required if clinically suspicious of bone metastasis. Besides, three gold fiducial markers will be inserted into the prostate under trans-rectal ultrasound (TRUS). The markers will be inserted in different planes within the prostate in order to facilitate the visualization of these markers in the on-board imaging at antero-posterior and lateral position.

5.0 Radiotherapy

1. Treatment planning and delivery

A. Patient set-up

- Patients will be positioned supine in a comfortable posture with Vaclock. Patients will be asked to drink 400cc water 1 hour before the simulation and each treatment. Patients will be asked to empty the rectum before the simulation and each treatment. Dulcolax will be given and will be used when necessary for the purpose of emptying the rectum. Patients will be advised to adhere to a low gas, low motility diet commencing one day prior to the treatment. The use of rectal balloons will be allowed but not mandatory.

B. Simulation/planning CT

- CT will be the primary image platform for treatment planning. The simulation should be performed in the supine treatment position, with the fiducial markers/rectal balloon in place (where utilized). Axial cuts of 3 mm or less will be acquired throughout the pelvis and prostate from the top of the iliac crests superiorly to the perineum inferiorly.

C. Target volumes

Arm 1

- The clinical target volume (CTV1) will be the whole prostate as defined by the non-contrast axial planning CT scan for the low risk group of patients. For intermediate risk group of patients, apart from CTV1, another clinical target volume(CTV2) will be

the whole prostate plus proximal two-third of seminal vesicles. MRI will be co-registered with the planning CT scan to aid for target contouring. The planning target volume (PTV) will be defined as the CTV plus 10 mm margin posteriorly and 6 mm in all other direction. To meet dose constraints, if necessary, the anterior margin can be reduced to 5mm.

<u>Arm 2</u>

- The clinical target volume (CTV1) will be the whole prostate as defined by the non-contrast axial planning CT scan for the low risk group of patients. For intermediate risk group of patients, apart from CTV1, another clinical target volume (CTV2) will be the whole prostate plus proximal two-third of seminal vesicles. MRI will be co-registered with the planning CT scan to aid for target contouring. The planning target volume (PTV) will be defined as the CTV plus 3 mm margin posteriorly and 5 mm in all other direction. To meet dose constraints, if necessary, the anterior margin can be reduced to 3 mm.

D. Critical structures

- The normal tissue volume to be contoured will include bladder, rectum, bilateral femora (to the level of ischial tuberosity) and penile bulb. The bladder should be contoured from its base to the dome. The rectum should be contoured from the anus (at the level of the ischial tuberosities) for a length of 15 cm or to the rectosigmoid flexure. This generally is below the bottom of the sacroiliac joints.

E. Dosimetry

- Total prescribed dose

<u>Arm 1</u>

- ■ Patients will receive 38 fractions of radiation, each fraction size will be 2Gy. The total dose will be 78Gy to PTV 1. Whereas the total dose will be 70Gy over 38 fractions to PTV 2. The treatment will be delivered 5 fractions per week consecutively except public holiday, and the total duration of treatment will be 7.5 to 8 weeks.

<u>Arm 2</u>

- ■ Patients will receive 5 fractions of radiation; each fraction size will be 7.25Gy. The total dose will be 36.25 Gy to PTV1. Whereas the total dose will be 32.5Gy over 5 fractions to PTV2. The 5 treatments will be scheduled to be delivered twice a week over approximately 15-17 days. A minimum of 72 hours and a maximum of 96 hours should separate each treatment. No more than 2 fractions will be delivered per week. The total duration of treatment will be no shorter than 15 days and no longer than 17 days.

- Dose coverage
  - ■ The isodose line used for the prescription dose should cover a minimum of 95%of the PTV.
- Minimum dose
  - ■ The minimum dose within the PTV to a point that is 0.03 cc in size must be ≥95% of the prescribed dose.
- Planning technique
  - ■ RapidArc plans were generated using double arcs, operating two complementary coplanar arcs of 350° (one counter-clockwise from 175° to

185°, one clockwise from 185° to 175°). Two arcs were used in order to achieve dose distributions with higher dose homogeneity in the targets, and lower maximum and mean doses to the OARs. The field size was set manually to include the whole tumor 3-dimensionally and the collimator was twisted 20 degrees to reduce the tongue-and-groove effect to the organs next to the target. Dose intensity modulation was achieved by the continuous variation of the gantry speed, dose rate and MLC positions. RA plan optimization was based on the Progressive Resolution Optimization (PRO) algorithm using five levels of optimization at a total of 177 checkpoints.

F. Dose constraint/specification

Arm 1

PTV	Dmin ≥ 95% of prescribed dose<br>≤3% received >107% of prescribed dose<br>≤3% received <93% of prescribed dose
rectum	V75Gy ≤ 15%<br>V70Gy ≤ 20%<br>V65Gy ≤ 25%<br>V60Gy ≤ 35%<br>V50Gy ≤ 50%
bladder	V80Gy ≤ 15%<br>V75Gy ≤ 25%<br>V70Gy ≤ 35%<br>V65Gy ≤ 50%
femoral head	V50Gy ≤ 50%
penile bulb (recommended)	D70 ≤ 70Gy<br>D90 ≤ 50Gy
normal tissue	Dmax≤ 110% of prescribed dose

Arm 2

Organ	Volume	Dosimetry parameters
Prostate (PTV1)	Maximum point dose (1cc)	≤38.78Gy
	Minimum dose received by 95% of PTV	≥ 36.25 Gy
	Minimum dose received by PTV	≥ 34.4 Gy
Prostate (PTV2)	Maximum point dose (1cc)	≤38.78Gy
	Minimum dose received by 95% of PTV	≥ 32.5Gy
	Minimum dose received by PTV	≥ 30.9Gy
Rectum	Maximum point dose (1cc)	≤ 38.06 Gy
	Less than 3 cc	< 34.4 Gy
	90% rectum	≤ 32.625 Gy
	80% rectum	≤29 Gy

	50% rectum	≤18.125 Gy
Bladder	Maximum point dose (1cc)	≤38.06 Gy
	90% Bladder	≤ 32.625 Gy
	50% Bladder	≤18.125 Gy
Penile bulb (recommended)	Maximum point dose	No more than 100% of prescription dose
	Less than 3 cc	20 Gy
Femoral heads Skin (recommended)	Less than 10 cc cumulative (both sides)	20 Gy
	Maximum point dose	30 Gy

G. Image-guidance for target localization

- After patient is set up on the treatment table, 2D (on-board imaging) and 3D CT (Cone-beam CT) systems that use x-rays will be used to align the patient with the treatment machine geometry based on the treatment plan. The alignment result will be evaluated by the attending physician and attending physicist and be approved for treatment by attending physician. The alignment data will be recorded. A rectal balloon can be used to immobilize the prostate.
- The initial localizations and alignment is based on the center of mass of the fiducial markers. Significant rotations may be corrected at initial localization stage, and intra-fractional rotations will be ignored. Further adjustment during the treatment will be translational shift of the center of mass determined via IGRT technique, using remote couch motion unless an institution has the ability to correct for rotations.
- For any image-guidance procedure, comparison with reference images or baseline data should be performed and reviewed by attending physician. The comparison can be done both manually and automatically. For any image-guidance method, if any deviation is larger than 2 mm, correction should be performed.

2. Supportive measures

A. Urinary
- Symptomatic urinary medicines, (e.g. tamsulosin) are allowed at the discretion of the treating radiation oncologist or urologist.

B. Bladder
- Patients will be asked to drink 400cc water 1 hour before the simulation and each treatment.

C. Bowel
- Patients will be asked to empty the rectum before the simulation and each treatment. Dulcolax will be given and will be used when necessary for the purpose of emptying the rectum. Patients will be advised to adhere to a low gas, low motility diet commencing one day prior to the treatment.

3. <u>Radiation therapy adverse events</u>

- All patients will be seen weekly by their radiation oncologist during radiation therapy. Any observations regarding radiation reactions will be recorded and should include attention to the following potential side effects:
  - Small bowel or rectal irritation manifesting as abdominal cramping, diarrhea, rectal urgency,proctitis, or hematochezia;
  - Bladder complications including urinary frequency/urgency, dysuria, hematuria, urinary tract infection, and incontinence;
  - Radiation dermatitis.
- Clinical discretion may be exercised to treat side effects from radiation therapy. Diarrhea/rectal frequency/urgency may be managed with diphenoxylate or loperamide. Bladder irritation may be mitigated with phenazopyridine. Urinary frequency/urgency can be managed with anticholinergic agents or alpha-blockers such as tamsulosin. Erectile dysfunction can be managed with phosphodiesterase (PDE) inhibitors such as sildenafil.
- Adverse Events (AEs) and Serious Adverse Events (SAEs) Reporting Requirements
  - **Definition of an AE**: Any unfavorable and unintended sign (including an abnormal laboratory finding), symptom, or disease temporally associated with the use of a medical treatment or procedure regardless of whether it is considered related to the medical treatment or procedure(attribution of unrelated, unlikely, possible, probable, or definite). [CTEP, NCI Guidelines: Adverse Event Reporting Requirements. January 2005;
  - **Definition of an SAE**: Any adverse experience occurring during any part of protocol treatment and 30 days after that results in any of the following outcomes:
    - Death;
    - A life-threatening adverse experience;
    - Inpatient hospitalization or prolongation of existing hospitalization;
    - A persistent or significant disability/incapacity;
    - A congenital anomaly/birth defect.

- Important medical events that do not result in death, are not life threatening, or do not require hospitalization may be considered an SAE, when, based upon medical judgment, they may jeopardize the patient and may require medical or surgical intervention to prevent one of the outcomes listed in the definition. Any pregnancy occurring on study must be reported via CCTU as a medically significant event.

6.0 Hormonal therapy

Neoadjuvant androgen deprivation (ADT) will be given to intermediate risk patients (T2b-c or GS 7 or PSA 10-20) before the start of radiotherapy. Two injections of ADT (every 3 months) will be given prior radiotherapy and the radiotherapy will be started 1 month after the last injection of ADT.

7.0 Patient assessment

A. Pre-treatment evaluation

- PSA should be obtained for at least 10 days after prostate biopsy

- Completion of the EPIC is mandatory for all patients.

B. Evaluation during the treatment
- Patients will be seen and evaluated at least weekly during radiation therapy with documentation of tolerance, including acute reactions.
- Delay in treatment is discouraged unless the patient's medical condition or side-effects of treatment merit a delay. Delay of treatment will be at the discretion of the treating radiation oncologist.

C. Evaluation following treatment
- After the second year (24 months) following radiation, follow-up will continue every 6 months for years 3, 4, and 5; then annually thereafter.
- EPIC will be perform at 3, 6, 9, 12, 18 and 24 months after completion of radiotherapy.
- A needle biopsy is encouraged: from the site of original tumor within the prostate and/or other site of original tumor identified by the transrectal ultrasound, as indicated for rising PSA or clinical failure
- A bone scan will be performed as clinically indicated, e.g., if the patient develops a PSA recurrence with a rapid doubling time (< 6 months) or if the patient develops symptoms suggesting the presence of metastatic disease.

D. Criteria for biochemical recurrence
- Biochemical (PSA) recurrence is defined according to the proposed new Radiation Therapy Oncology Group/American Society for Therapeutic Radiology and Oncology (RTOG-ASTRO)criteria also known as the RTOG Phoenix definition: an increase of the PSA level at least 2ng/mL greater than the minimum level reached after therapy (lowest PSA+ 2 criterion). All PSA levels done during a follow-up interval will be recorded on the data forms.

E. Criteria for nodal recurrence
- Clinical criteria for local recurrence are progression (increase in palpable abnormality) at anytime, failure of regression of the palpable tumor by 2 years, and redevelopment of a palpable abnormality after complete disappearance of previous abnormalities. Needle biopsy is recommended. The presence of palpable disease must be recorded on the data collection forms for initial and follow-up evaluations of the patient.
- Histologic criteria for local recurrence are presence of prostatic carcinoma upon biopsy and positive biopsy of the palpably normal prostate more than 2 years after the start of treatment.

F. Criteria for non-local recurrence
- Distant metastasis will be documented if clinical or bone scan evidence is demonstrated. Ultrasound evaluation of the prostate with needle biopsy as indicated by the findings is recommended at the time distant metastasis is reported.
- Regional metastasis will be documented if there is radiographic evidence (CT or MRI) of lymphadenopathy and histologic confirmation.

8.0 <u>Statistical consideration</u>

1. Primary endpoint
- Evaluate and compare the HRQOL by the proportion of patients with > 5-point and > 2-point reductions in the EPIC bowel and urinary domains, respectively, at 1 year

compared with baseline, between the two treatment arms.

2. Secondary endpoint
- Rate of acute and late GI and GU toxicity for each arm at 1, 2, and 5 years
- Biochemical failure-free survival
- Disease-specific survival
- Overall survival

3. Sample size derivation and accrual
- The primary goal of this phase II study is to comparing the SBRT and IMRT. The choice of regimen will be based on maintaining acceptable patient reported HRQOL from baseline. There will be two co-primary endpoints based on summary scores from the bowel and urinary domains of the EPIC questionnaire. Although HRQOL is collected at multiple time points, it has been decided that EPIC information collected at 1 year will be compared to baseline EPIC data to calculate the sample size and to test the primary hypothesis. In part, this is due to missing data issues with patient reported outcome data and it is more likely to have full HRQOL patient information earlier in the study (e.g., 6 months, 1 year) than later (e.g., 2 years on). The hypothesis for each arm is that the probability of the change in EPIC HRQOL scores (1-year minus baseline) is less than (or equal to) an acceptable limit. Thus, for each arm, the hypotheses are:

$$H_0 : p \le p_0 \quad v.s. \quad H_1 : p > p_a$$

where p_0 is the probability under the null hypothesis.

From the conventional arm of RTOG 0415, in an analysis of 108 patients (mean pretreatment score 94 with SE=9 and range 61-100), 38 patients (35%) had a change in EPIC bowel domain score (baseline to 1-year) that was worse than 5 points. For 110 patients (mean pretreatment score 88, SE=12, range 35-100), 43 (39%) had a change in EPIC urinary domain score that was worse than 2 points. Mean reduction in bowel and urinary scores have also been published in a historical series by Wei et al. (2002). The mean reduction in bowel and urinary scores observed in the RTOG 0415 analysis are in keeping with the series published by Wei. Standard radiotherapy in regimens such as used in the conventional arm of 0415 is well tolerated. Thus the HRQOL analysis from the 0415 study will be used in the sample size calculation. The percentage of patients with change in EPIC bowel domain score (baseline to 1-year) that was worse than 5 points and a change in EPIC urinary domain score that was worse than 2 points are felt to be clinically meaningful endpoints to assess for tolerability and safety.

A rate for the worse-than-5 point change in bowel score of up to 35% of patients will be considered acceptable, with a rate ≥60% specified as unacceptable. Similarly, a rate for the worse-than-2 point change in urinary score of up to 40% will be considered acceptable, with a rate ≥65% unacceptable. Using the normal approximation to the binomial distribution for p, the formula to calculate the sample size is given by:

$$n=\left[\frac{z_{1-\alpha}\sqrt{p_0(1-p_0)}+z_{1-a}\sqrt{p_a(1-p_a)}}{(p_0-p_a)}\right]^2$$

where p_0 is as previously defined, pa= the alternative probability, z_q=100(qth)percentile of the standard normal distribution. With the aforementioned design parameters, a sample size of 60 eligible patients (30 per arm) is required with 80% power and significance level 0.025 (one-sided test). The significance level 0.025 will be used for each endpoint in order to preserve an overall significance level of 0.05. Adjusting for 5% ineligibility, **the required sample size is 64 patients (32 per arm).**

4. Analysis of primary endpoint

- The co-primary endpoints are: (1) the proportion of patients with change from baseline to the 1-year EPIC bowel domain score that exceeds 5 points; and (2) the proportion of patients change from baseline to 1-year EPIC urinary domain score that exceeds 2 points. Analysis will be done separately for each arm. The average score of each arm will be calculated, along with the standard error, based on the hypothesis

$$H_0 : p \le p_0 \quad v.s. \quad H_1 : p > p_a$$

- The one-sample z-test for proportion with significance level of 0.025 (for each endpoint) will be used to test the hypothesis.
- For a given arm, if H_0 is rejected for either EPIC domain, then it will be concluded that the regimen given on that arm is unacceptable in terms of PRO. However if it is not rejected for both domains, then that regimen will be considered acceptable. If both arms yield an acceptable result, then consideration may be given to using other factors, specifically, results of secondary endpoints to decide if one regimen is more acceptable than the other one. In each arm, logistic regression will be performed, both an unadjusted and adjusted analysis. In the adjusted analysis, the variables in the regression will at least include the stratification variable, treatment modality, and, if appropriate, additional variables such as Gleason score, PSA, age and race can be included.

5. Analysis of secondary endpoints

a. Adverse events are evaluated by the CTEP Active Version of the NCI Common Terminology Criteria for Adverse Events (CTCAE). The treatment-related attribution includes definitely, probably or possibly related to treatment. An acute adverse event is defined as the first occurrence of worst severity of the adverse event ≤30 days after the completion of RT.A late adverse event is defined as the first occurrence of worst severity of adverse event >30days after RT completion.

b. Biochemical failure-free survival

- Biochemical failure-free survival will be measured from the date of registration to the date of documentation of biochemical recurrence (RTOG phoenix definition) or until the date of death from any cause. DFS at 1, 2,and 5 years will be estimated by the Kaplan-Meier method. Also, 95% confidence intervals will be reported.

c. Disease-specific survival

- The disease-specific survival duration will be measured from the date of registration to the date of documentation of death due to prostate cancer or until the date of death from any cause. DSS at 1, 2,and 5 years will be estimated by the Kaplan-Meier method. Also, 95% confidence intervals will be reported.

d. Overall survival

- The overall survival duration will be measured from the date of registration to the date of documentation of death from any cause. OS at 1, 2,and 5 years will be estimated by the Kaplan-Meier method. Also, 95% confidence intervals will be reported.

9.0 Data and protocol management

The data management will be conducted in compliance with the protocol, the International Conference on Harmonisation of Good Clinical Practice (ICH GCP) guidelines and the Declaration of Helsinki. The protocol will be adhered and complied to the principle of ICH-GCP.

All collected data will be saved in electronic database securely and confidentially with password protected. Private information (i.e. name, address, identity number etc) will not be included in database and collected information will only be identified by an anonymous code for each patient. Authority to access private information will only be provided to the respective investigators and their designee. It is the responsibility of each investigator to manage the collected data securely and to protect private information.

10.0 Publication Policy

The study results shall be presented at international academic conferences and the final report shall be submitted for publication in peer reviewed international journal. This will be determined by the principle investigator. Prior to any submission for presentation or publication, an approval should be obtained from the principal investigators. Supporting groups should be acknowledged.

Key references

1. Brenner DJ, Hall EJ. Fractionation and protraction for radiotherapy of prostate carcinoma. *Int J RadiatOncolBiolPhys.* 43: 1095-1101, 1999.
2. Brenner DJ, Martinez AA, Edmundson GK*, et al.* Direct evidence that prostate tumors show high sensitivity tofractionation (low alpha/beta ratio), similar to late-responding normal tissue. *Int J RadiatOncolBiol Phys.* 52: 6-13, 2002.
3. Dale RG, Jones B. Is the alpha/beta for prostate tumors really low? In regard to Fowler et al., IJROBP2001;50:1021-1031. *Int J RadiatOncolBiol Phys.* 52: 1427-1428, author reply 1428, 2002.
4. Fowler JF, Ritter MA. A rationale for fractionation for slowly proliferating tumors such as prostatic adenocarcinoma.*Int J RadiatOncolBiol Phys.* 32: 521-529, 1995.
5. Fowler JF, Ritter MA, Chappell RJ*, et al.* What hypofractionated protocols should be tested for prostate cancer?*Int J RadiatOncolBiol Phys.* 56: 1093-1104, 2003.
6. King CR, Brooks JD, Gill H*, et al.* Stereotactic Body Radiotherapy for Localized Prostate Cancer: Interim Resultsof a Prospective Phase II Clinical Trial. *Int J RadiatOncolBiolPhys*2008.
7. King CR, Lehmann J, Adler JR*, et al.* CyberKnife radiotherapy for localized prostate cancer: rationale andtechnical feasibility. *Technol Cancer Res Treat*. 2: 25-30, 2003.
8. Lukka H, Hayter C, Julian JA*, et al.* Randomized trial comparing two fractionation schedules for patients withlocalized prostate cancer. *J ClinOncol*. 23: 6132-6138, 2005.
9. Madsen BL, Hsi RA, Pham HT, et al: Stereotactic hypofractionatedaccurate radiotherapy of the prostate (SHARP), 33.5 Gy in five fractionsfor localized disease: First clinical trial results. Int J RadiatOncolBiolPhys 67:1099-1105, 2007
10. Friedland JL, Freeman DE, Masterson-McGary ME, et al: Stereotacticbody radiotherapy: An emerging treatment approach for localized prostatecancer. Technol Cancer Res Treat 8:387-392, 2009
11. Katz AJ, Santoro M, Ashley R, et al: Stereotactic body radiotherapy fororgan-confined prostate cancer. BMC Urol 10:1, 2010
12. McBride SM, Wong DS, Dombrowski JJ, et al: Hypofractionated stereotacticbody radiotherapy in low-risk prostate adenocarcinoma: Preliminaryresults of a multi-institutional phase 1 feasibility trial. Cancer118:3681-3690, 2012
13. Boike TP, Lotan Y, Cho LC, et al: Phase I dose-escalation study ofstereotactic body radiation therapy for low- and intermediate-risk prostatecancer. J ClinOncol 29:2020-2026, 2011

Appendix I

	Pre-study entry				During treatment	Follow-up (months)<br>Every 6 months for years 3, 4, and 5, then annually							
	≤60days	≤90days	≤180days	≤365days	Weekly during RT	3	6	9	12	15	18	21	24
History/physical exam including digital rectal examination (DRE)	X												
Physical exam including DRE						X	X	X	X	X	X	X	X
Performance status	X				X	X	X	X	X	X	X	X	X
Prostate biopsy with Gleason score				X									
PSA	X					X	X	X	X	X	X	X	X
Testosterone			X										
Adverse event evaluation					X	X	X	X	X	X	X	X	X
MRI pelvis	X												X*
Bone scan	X*												X*
EPIC questionnaire	X					X	X	X	X		X		X
* if clinically indicated													

Appendix II

ZUBROD PERFORMANCE SCALE

0 Fully active, able to carry on all predisease activities without restriction

1 Restricted in physically strenuous activity but ambulatory and able to carrywork of a light or sedentary nature. For example, light housework, officework

2 Ambulatory and capable of all self-care but unable to carry out any workactivities. Up and about more than 50% of waking hours

3 Capable of only limited self-care, confined to bed or chair 50% or more ofwaking hours

4 Completely disabled. Cannot carryon self-care. Totally confined tobed

5 Death

APPENDIX III

AJCC STAGING SYSTEM
PROSTATE, 7th Edition
DEFINITIONS OF TNM

Source: Edge, SB, ed. *AJCC Cancer Staging Manual*. 7th ed. New York, NY: Springer; 2010.

Primary Tumor, Clinical (T)
TX Primary tumor cannot be assessed
T0 No evidence of primary tumor
T1 Clinically inapparent tumor neither palpable nor visible by imaging
T1a Tumor incidental histologic finding in 5% or less of tissue resected
T1b Tumor incidental histologic finding in more than 5% of tissue resected
T1c Tumor identified by needle biopsy (e.g., because of elevated PSA)

T2 Tumor confined with prostate*
T2a Tumor involves one-half of one lobe or less
T2b Tumor involves more than one-half of one lobe but not both lobes
T2c Tumor involves both lobes

T3 Tumor extends through the prostate capsule**
T3a Extracapsular extension (unilateral or bilateral)
T3b Tumor involves the seminal vesicle(s)

T4 Tumor is fixed or invades adjacent structures other than seminal vesicles such as externalsphincter, rectum, bladder, levator muscles and/or pelvic wall
*Note: Tumor found in one or both lobes by needle biopsy, but not palpable or reliably visible by imaging, isclassified as T1c
**Note: Invasion into the prostatic apex or into (but not beyond) the prostatic capsule is classified not as T3 butas T2.

Primary Tumor, Pathologic (pT) *
pT2 Organ confined
pT2a Unilateral, one-half of one side or less
pT2b Unilateral, involving more than one-half of side but not both sides
pT2c Bilateral disease

pT3Extraprostatic extension
pT3aExtraprostatic extension or microscopic invasion of bladder neck**
pT3b Seminal vesicle invasion

pT4 Invasion of rectum, levator muscles, and/or pelvic wall
*Note: There is no pathologic T1 classification
**Note: Positive surgical margin should be indicated by an R1 descriptor (residual microscopic disease).

Regional Lymph Nodes (N)
Clinical
NX Regional lymph nodes were not assessed
N0 No regional lymph node metastasis
N1 Metastasis in regional lymph node(s)
Pathologic
pNX Regional nodes not sampled
pN0 No positive regional nodes
pN1 Metastases in regional node(s)

Distant Metastasis (M)*
M0 No distant metastasis
M1 Distant metastasis
M1a Nonregional lymph node(s)
M1b Bone(s)
M1c Other site(s) with or without bone disease
*Note: When more than one site of metastasis is present, the most advanced category is used;
pM1c is most advanced.

Histologic Grade (G)
Gleason X Gleason score cannot be processed
Gleason ≤6 Well-differentiated (slight anaplasia])
Gleason 7 Moderately differentiated (moderate anaplasia)
Gleason 8-10 Poorly differentiated/undifferentiated (marked anaplasia)

Appendix IV

EPIC

The Expanded Prostate Cancer Index Composite
擴展前列腺癌綜合指數

這份問卷的設計，是為了衡量前列腺癌症病人，在生活質素方面的各項問題。為幫助我們得到最準確的量度數據，請你如實完整地回答所有問題，這是至為重要的。

請緊記，正如所有醫療紀錄一樣，這份問卷□所載的資料，會嚴格加以保密。

今日的日期 (請填寫你完成問卷的日期): 年________月________日________

姓名 (可選擇不填寫): ____

出生日期 (可選擇不填寫): 年__________月__________日__________

請勿在
此欄書寫

排尿功能

這部份是關於你的排尿習慣。**請只根據過去的4個星期**的情況回答。

1. 在過去的**4個星期**，你漏尿的情況，有多頻密？

每天超過一次	**1**		
每天大約一次	**2**		
每星期超過一次	**3**	**(請圈出一個數字)**	**23/**
每星期大約一次	**4**		
絕少發生或沒有漏尿	**5**		

2. 在過去的**4個星期**，你小便有血的情況，有多頻密？

每天超過一次	**1**		
每天大約一次	**2**		
每星期超過一次	**3**	**(請圈出一個數字)**	**24/**
每星期大約一次	**4**		
絕少發生或小便沒有血	**5**		

3. 在過去的**4個星期**，你在排尿時感到疼痛或灼熱的情況，有多頻密？

每天超過一次	**1**		
每天大約一次	**2**		**25/**
每星期超過一次	**3**	**(請圈出一個數字)**	
每星期大約一次	**4**		
絕少發生或排尿時沒有感到疼痛或灼熱	**5**		

4. 在過去的**4個星期**，以下哪個答案最能形容你控制排尿的情況？

完全不能控制排尿	**1**		
經常滴尿	**2**		**26/**
偶然滴尿	**3**	**(請圈出一個數字)**	
完全可以控制排尿	**4**		

EPIC 2.2002 2002年版權所有 密芝根大學 (University of Michigan) All rights reserved 保留一切版權

請勿在
此欄書

5. 在過去的**4個星期,**你通常每日要用多少塊衛生墊或成人尿片，以控制漏尿？

**1**塊也不用......	**0**		
每天**1**塊......	**1**		
每天**2**塊......	**2**	**(**請圈出一個數字**)**	
每天**3**塊或以上......	**3**		**27/**

6. 在過去的**4個星期，**如果你有以下任何情況，那麼以下每一個情況對你造成的問題有多大？

(請於下列**a**至**f**每一行都圈出一個數字**)**

	沒有問題	很小問題	小問題	中問題	大問題	
**a.** 滴尿或漏尿......	**0**	**1**	**2**	**3**	**4**	**28/**
**b.** 排尿時感到疼痛或灼熱......	**0**	**1**	**2**	**3**	**4**	**29/**
**c.** 排尿時出血......	**0**	**1**	**2**	**3**	**4**	**30/**
**d.** 排尿弱或不能徹底排尿......	**0**	**1**	**2**	**3**	**4**	**31/**
**e.** 睡眠中因為要排尿而起床......	**0**	**1**	**2**	**3**	**4**	**32/**
**f.** 日間小便頻頻......	**0**	**1**	**2**	**3**	**4**	**33/**

7. 整體來說，在過去的**4個星期，**你的排尿功能對你構成多大問題？

沒有問題......	**1**		
很小問題......	**2**		
小問題......	**3**	**(**請圈出一個數字**)**	**34/**
中問題......	**4**		
大問題......	**5**		

2002年版權所有 密芝根大學 (University of Michigan) All rights reserved 保留一切版權

請勿在
此欄書寫

排便習慣

以下部份是關於你的排便習慣和腹痛。
請只根據過去的4個星期的情況回答。

8. 在過去**4個星期，**你直腸有急迫感**(**即感到想去排便，但卻沒有大便**)** 的情況, 有多頻密？

每天超過一次.................................. 1
每天大約一次 2
每星期超過一次 .... 3 **(**請圈出一個數字**)** **42/**
每星期大約一次.............................. 4
絕少發生或沒有這個問題............... 5

9. 在過去的**4個星期，**你不能控制大便或糞便漏出的情況，有多頻密？

每天超過一次................................. 1
每天大約一次................................. 2
每星期超過一次............................. 3 **(**請圈出一個數字**)** **43/**
每星期大約一次............................. 4
絕少發生或沒有控制大便的問題.... 5

10. 在過去的**4個星期，**你的大便稀爛或呈液體狀**(**不成形、水狀、糊狀**)** 的情況，有多頻密？

大便沒有稀爛或呈液體狀............... 1
絕少發生... 2
約佔半數時間................................. 3 **(**請圈出一個數字**)** **44/**
通常如此 4
總是這樣... 5

11. 在過去的**4個星期，**你排便出血的情況，有多頻密？

排便沒有出血................................. 1
絕少發生... 2
約佔半數時間................................. 3 **(**請圈出一個數字**)** **45/**
通常如此 4
總是這樣... 5

EPIC 2.2002 2002年版權所有 密芝根大學 (University of Michigan) All rights reserved 保留一切版權

請勿在
欄書寫

性功能

以下內容是關於你目前的性功能和性滿足感。許多問題是非常個人的，但卻可以幫助我們明白你每天面對的重要困難。請緊記，**這份問卷的資料，會完全保密**，請只按你**過去4個星期**的情況，如實回答。即使你完全沒有性生活，也請你盡力回答以下的問題。

17. 在**過去的4個星期**，你怎樣為以下各項評分？ **(每一行均請圈出一個數字)**

	極差至沒有	差	普通	好	甚好	
a. 你的性慾程度？	1	2	3	4	5	56/
b. 你勃起的能力？	1	2	3	4	5	57/
c. 你能達到高潮的能力？	1	2	3	4	5	58/

18. 在**過去的4個星期**，你怎樣形容勃起的狀態？

完全沒有勃起 1
堅挺度不足以進行任何性活動 2
堅挺度僅足以進行自慰和性愛前奏 ..... 3 **(請圈出一個數字)** 59/
堅挺度足以性交 4

19. 在**過去的4個星期**，你怎樣形容勃起的**頻密程度**？

當我想勃起時，完全不能做到 1
當我想勃起時，只有少於半數情況做到 2
當我想勃起時，約有半數情況做到 ..... 3 **(請圈出一個數字)** 60/
當我想勃起時，約有多於半數情況做到 4
每當我想勃起時，我都做得到 5

20. 在**過去的4個星期**，在早上或晚間醒來時有勃起的情況，有多頻密？

完全沒有 1
少於一星期一次 2
約一星期一次 ..... 3 **(請圈出一個數字)** 61/
一星期數次 4
每天都有 5

EPIC 2.2002 2002年版權所有 密芝根大學 (University of Michigan) All rights reserved 保留一切版權

請勿在此
欄書寫

21. 在過去的**4個星期，**你進行任何與性事有關(性交或非性交)的活動，有多頻密？

完全沒有	1		
少於一星期一次	2		
約一星期一次	3	**(請圈出一個數字)**	**62/**
一星期數次	4		
每天都有	5		

22. 在過去的**4個星期，**你進行性交有多頻密？

完全沒有	1		
少於一星期一次	2		
約一星期一次	3	**(請圈出一個數字)**	**63/**
一星期數次	4		
每天都有	5		

23. 整體來說，在過去的**4個星期，**你怎樣為你的性能力評分？

極差	1		
差	2		
普通	3	**(請圈出一個數字)**	**64/**
好	4		
極好	5		

24. 在過去的**4個星期，**如果你有以下任何情況，那麼以下每一個情況造成的問題有多大？

(請於下列a至c每一行都圈出一個數字)

	沒有問題	很小問題	小問題	中問題	大問題	
a. 你的性慾程度	0	1	2	3	4	**65/**
b. 你勃起的能力	0	1	2	3	4	**66/**
c. 你能達到高潮的能力	0	1	2	3	4	**67/**

25. 整體來口，在過去的**4個星期，**你的性功能或缺乏性功能對你造成的問題有多大？

沒有問題	1		
很小問題	2		
小問題	3	**(請圈出一個數字)**	**68/**
中問題	4		
大問題	5		

EPIC 2.2002 2002年版權所有 密芝根大學 (University of Michigan) All rights reserved 保留一切版權

賀爾蒙功能
部份病人會因為醫生使用**賀爾蒙**治療，而引致身體突然出汗發熱，乳頭有硬塊或觸痛，情緒低落或抑鬱，身體極度疲倦，或體重增加。以下部份就是關於上述的問題。請只根據**過去4個星期**的情況回答。

請勿在此欄書寫

26. 在**過去的4個星期**，如果你有熱潮或陣熱 (即身體突然出汗發熱)，它們有多頻密？

每天超過一次	1		
每天大約一次	2		
每星期超過一次	3	(請圈出一個數字)	69/
每星期大約一次	4		
絕少發生或一次也沒有	5		

27. 在**過去的4個星期**，你胸部或乳頭位置觸痛的情況，有多頻密？

每天超過一次	1		
每天大約一次	2		
每星期超過一次	3	(請圈出一個數字)	70/
每星期大約一次	4		
絕少發生或一次也沒有	5		

28. 在**過去的4個星期**，你情緒感到抑鬱的情況，有多頻密？

每天超過一次	1		
每天大約一次	2		
每星期超過一次	3	(請圈出一個數字)	71/
每星期大約一次	4		
絕少發生或沒有感到抑鬱	5		

29. 在**過去的4個星期**，你感到缺乏精力的情況，有多頻密？

每天超過一次	1		
每天大約一次	2		
每星期超過一次	3	(請圈出一個數字)	72/
每星期大約一次	4		
絕少發生或身體沒有感到缺乏精力	5		

30. 在**過去的4個星期**，如果你的體重有變，變化是多少？

增加了10磅或以上	1		
增加了不足10磅	2		
體重沒有改變	3	(請圈出一個數字)	73/
減少了不足10磅	4		
減少了10磅或以上	5		

請勿在此
欄書寫

31. 在過去的4個星期，如果你有以下任何情況，那麼以下每一個情況對你造成的問題有多大？

(請於下列a至f每一行都圈出一個數字)

		沒有問題	很小問題	小問題	中問題	大問題	
a.	熱潮(身體突然出汗發熱)......	0	1	2	3	4	74/
b.	乳頭位置觸痛/增大..................	0	1	2	3	4	75/
c.	體毛脫落..................................	0	1	2	3	4	76/
d.	情緒低落或抑鬱......................	0	1	2	3	4	77/
e.	身體沒有精力..........................	0	1	2	3	4	78/
f.	體重改變..................................	0	1	2	3	4	79/

整體滿意程度

32. 整體來說，你對於你所接受的前列腺癌治療，有多滿意？

極不滿意..	1		
不滿意..	2		
不確定..	3	(請圈出一個數字)	80/
滿意..	4		
極滿意..	5		

謝謝!!

EPIC 2.2002 2002年版權所有 密芝根大學 (University of Michigan) All rights reserved 保留一切版權

請勿在
此欄書寫

12. 在過去的**4個星期，**你在排便時感到疼痛的情況，有多頻密？

排便並不感到疼痛............	**1**		
絕少發生............................	**2**		
約佔半數時間........................	**3**	**(請圈出一個數字)**	**46/**
通常如此	**4**		
總是這樣	**5**		

13. 在過去的**4個星期，**在正常日子裡，你每天排便多少次？

兩次或以下............................	**1**		
三次至四次............................	**2**	**(請圈出一個數字)**	**47/**
五次或以上............................	**3**		

14. 在過去的**4個星期，**你的下腹或直腸感到抽搐疼痛的情況，有多頻密？

每天超過一次........................	**1**		
每天大約一次........................	**2**		
每星期超過一次......................	**3**	**(請圈出一個數字)**	**48/**
每星期大約一次......................	**4**		
絕少發生或沒有這個問題....	**5**		

15. 在過去的**4個星期，**如果你有以下任何情況，那麼以下每一個情況對你造成的問題有多大？
(請於下列a至f每一行都圈出一個數字)

	沒有問題	很小問題	小問題	中問題	大問題	
**a.** 有急於排便的感覺...............	**0**	**1**	**2**	**3**	**4**	**49/**
**b.** 排便次數增加.......................	**0**	**1**	**2**	**3**	**4**	**50/**
**c.** 水狀糞便..............................	**0**	**1**	**2**	**3**	**4**	**51/**
**d.** 不能控制排便........................	**0**	**1**	**2**	**3**	**4**	**52/**
**e.** 糞便帶血..............................	**0**	**1**	**2**	**3**	**4**	**53/**
**f.** 下腹/直腸疼痛......................	**0**	**1**	**2**	**3**	**4**	**54/**

16. 整體來說，在過去的**4個星期，**你排便習慣對你構成的問題有多大？

沒有問題................................	**1**		
很小問題................................	**2**		
小問題....................................	**3**	**(請圈出一個數字)**	**55/**
中問題....................................	**4**		
大問題....................................	**5**		

EPIC 2.2002 2002年版權所有 密芝根大學 (University of Michigan) All rights reserved 保留一切版權
