# Supplementary material for: Prospective Randomized Phase II Study of Stereotactic Body Radiotherapy (SBRT) vs. Conventional Fractionated Radiotherapy (CFRT) for Chinese Patients with Early-Stage Localized Prostate Cancer
Source: Curr Oncol. 2021 Dec 22;29(1):27–37. doi: 10.3390/curroncol29010003 (PMC8774487; doi:10.3390/curroncol29010003)
Supplement: Supplementary file 1 [file curroncol-29-00003-s001.zip › Supplementary Methods.pdf]

Supplementary Method Details

EPIC Score Cutoffs

The Expanded Prostate Cancer Index Composite (EPIC) questionnaire is a 50-item measure designed to evaluate patient function and bother after prostate cancer treatment using a Likert scale with responses transformed to a scale of 0 to 100. Higher scores indicate better QOL. The instrument includes four separately validated domains: urinary, bowel, sexual and hormonal.

A rate for the worse-than-5-point change in bowel score of ≤ 35% of patients was considered acceptable, and ≥ 60% as unacceptable. Similarly, a rate for the worse-than-2-point change in urinary score of ≤ 40% was considered acceptable, and ≥ 65% as unacceptable. Regarding the sexual and hormonal scores, we considered drops of > 11 points and > 3 points, respectively, to be clinically significant. With these cut-offs, a rate of < 35% of patients was deemed acceptable for the EPIC sexual score, and a rate of ≥ 60% was unacceptable. For the EPIC hormonal domain, a rate of < 38% was considered acceptable, and a rate of ≥ 63% was unacceptable.

Using a one sample z-test, 64 patients were required for 80% power at a 1-sided significance level of 0.025 for each co-primary endpoint. To account for the 5% of ineligible cases, the target accrual was 68 patients.

Radiotherapy Procedures

For simulation and treatment with both conventional fractionated radiotherapy (CFRT) and stereotactic body radiotherapy (SBRT), patients were immobilized in supine position using a Vac-Lok cushion. Planning computed tomography (CT) images were acquired with axial slices of 1.5-mm thickness, and then imported into the Eclipse treatment planning system (Varian Medical Systems Inc., Palo Alto, CA). Patients were instructed to take an oral enema (bisacodyl) the night before simulation and to drink 400 mL of water 1 hour before the procedure, immediately after emptying their bladders. For image guidance, three fiducials were implanted transrectally in the prostate under ultrasound guidance by an interventional radiologist at least 7 days prior to the simulation CT. Endorectal balloons (ERBs; QLRAD Inc., Miami, FL) were inflated with 90 mL sterile water and inserted to a depth that situated just behind the prostate gland in patients allocated to the SBRT arm. Magnetic resonance images were co-registered with the planning CT scan to aid target contouring.

The structures outlined included the prostate, proximal two-thirds of the seminal vesicles (SVs), rectum (from the level of the anus to the sigmoid flexure), bladder, penile bulb and femoral heads. The whole

prostate was included in the clinical target volume (CTV-1) in low-risk patients. For the intermediate-risk patients, a second clinical target volume (CTV-2) comprising the whole prostate plus the proximal two-thirds of the SVs was added. Margins of 10 mm/5 mm in all directions (except 6 mm/3 mm posteriorly) were expanded from the CTVs as the planning target volume (PTVs) for CFRT/SBRT patients, respectively.

The photon energy was a conventional 10 MV beam with the flattening filter. Two to four arcs with an arc length of 300 – 350 degrees were used depending on the anatomy of the patient and the complexity of the arc intensity modulation. The dose rate was set at 600 MU/min. The treatment plans were generated and optimized using Varian Eclipse 13.6. The 3D volumetric dose was calculated using the anisotropic analytic algorithm, such that 95% of PTVs were to receive the corresponding dose prescriptions, and the minimum dose within the PTV to a point that is 0.03 cc in size must be ≥ 95% of the prescribed dose.
